# Supplementary material for: The Construction and Exploration of a Comprehensive MicroRNA Centered Regulatory Network in Foxtail Millet (Setaria italica L.)
Source: Front Plant Sci. 2022 May 6;13:848474. doi: 10.3389/fpls.2022.848474 (PMC9121102; doi:10.3389/fpls.2022.848474)
Supplement: Supplementary file 4 [file Data_Sheet_4.zip › Sit-miRNovel1_Seita.4G131500.1_3985_TPlot.pdf]

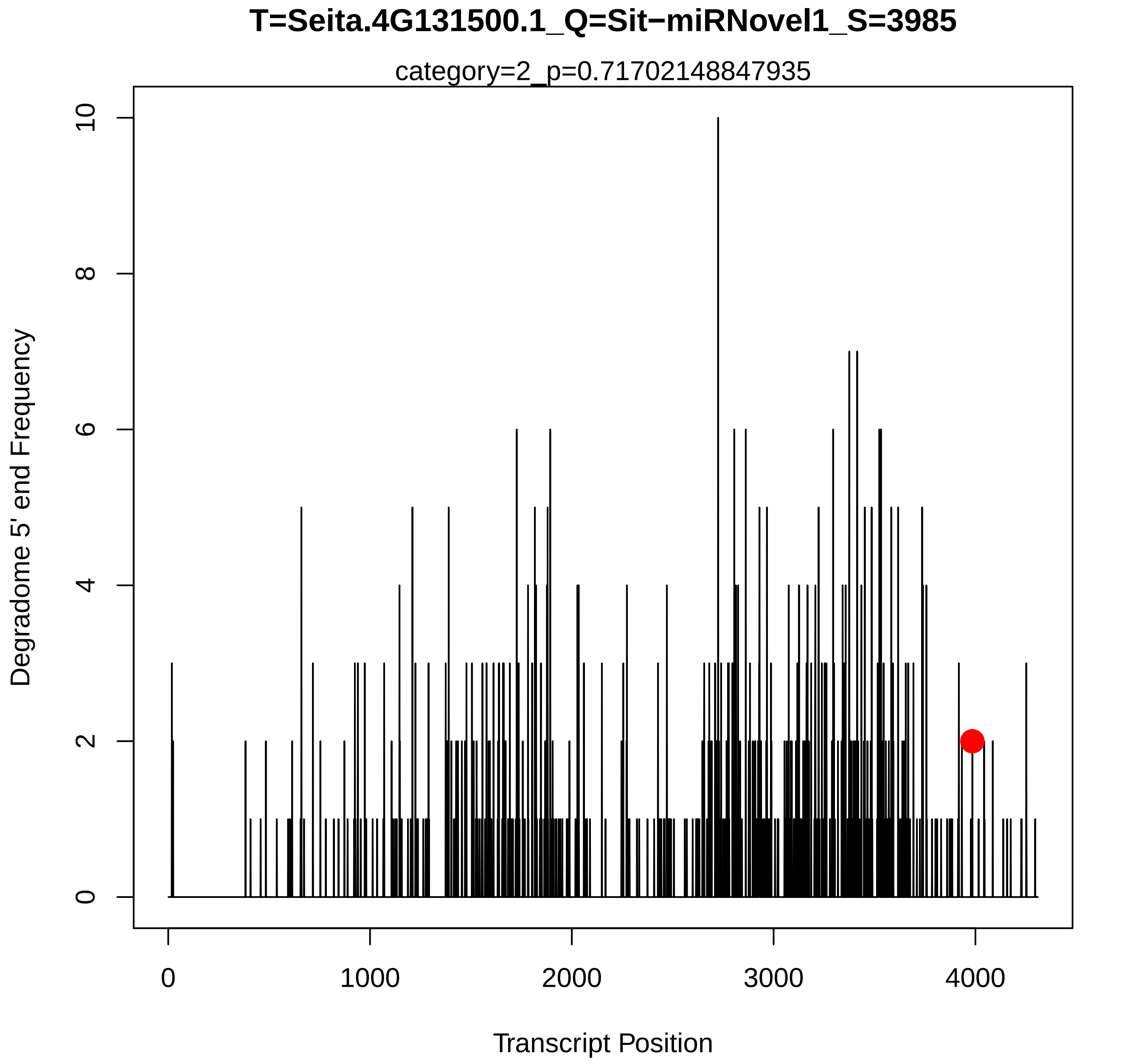
T=Seita.4G131500.1_Q=Sit−miRNovel1_S=3985
category=2_p=0.717021488847935
Degradome 5' end Frequency
Transcript Position
0
2
4
6
8
10
0
1000
2000
3000
4000
